# A framework to characterize the performance of early warning index alarm systems for patient monitoring

**DOI:** 10.1016/j.mex.2019.07.003

**Published:** 2019-07-08

**Authors:** Chathuri Daluwatte, Farid Yaghouby, Christopher Scully

**Affiliations:** a Division of Applied Regulatory Science, Office of Clinical Pharmacology, Center for Drug Evaluation and Research, U.S. Food and Drug Administration, United States; b Office of Science and Engineering Laboratories, Center for Devices and Radiological Health, U.S. Food and Drug Administration, United States

**Keywords:** A framework to characterize the performance of early warning index alarm systems for patient monitoring, Patient monitoring, Warning index, Alarm systems, Performance assessment

## Abstract

In [Scully, C.G., and Daluwatte, C., Evaluating performance of early warning indices to predict physiological instabilities. J Biomed Inform. 75 (2017) 14–21], a framework was presented to characterize the performance of warning indices to provide information on the 1) probability a critical health event will occur when a warning is given (analogous to positive predictive value) and 2) proportion of warned events to all events (analogous to sensitivity). This framework also provides information about the timeliness of the warnings with respect to event occurrence and the warning burden of the system.

• In the current work, we provide information on how this framework can be used when cases without events are present in a dataset to examine the proportion of warned non-events to all non-events (analogous to false positive rate).
• Information on steps to apply the method, software, data and results for the case study are also provided to enable implementation of the framework.
• Application and extension of the framework is demonstrated and discussed by adding non-event records to our previous case study comparing two warning strategies to predict physiologic instabilities.

## Graphical abstract

**Figure:**
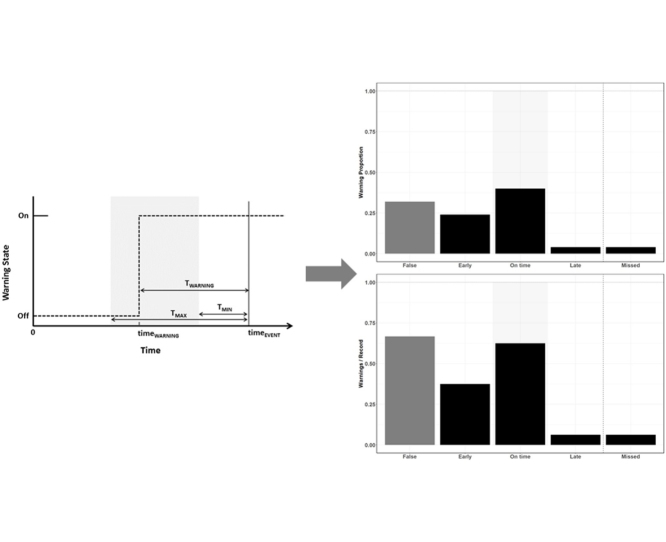

**Specifications Table**

**Table:** 

Subject Area:	Engineering
More specific subject area:	Multiparameter patient monitoring algorithms
Method name:	A Framework to Characterize the Performance of Early Warning Index Alarm Systems for Patient Monitoring
Name and reference of original method:	Scully, C.G., and Daluwatte, C., Evaluating performance of early warning indices to predict physiological instabilities. J Biomed Inform. 75 (2017) 14–21.
Resource availability:	Software, data and results for the case study provided as Supplemental materials.

## Method details

### Brief background

Multiparameter models that integrate physiological, laboratory and/or clinical data to provide an early warning of patients progressing towards a critical event have the potential to improve physiological monitoring in applications from adult and pediatric intensive care units [[1], [2], [3], [4]] to telemedicine [5,6]. The utility of an early warning index to indicate an event of interest may be affected by characteristics such as 1) probability the event occurs when a warning is given, 2) probability to correctly detect true events and 3) probability to incorrectly detect non-events as events. Traditionally this information is estimated using 1) positive predictive value, 2) sensitivity, and 3) false positive rate (also known as type I error and related to specificity as: false positive rate = 1 – specificity), respectively considering an alarm (occurring at some point prior to the event on event records) per record. However, in continuous physiological monitoring the timeliness of the warning also needs to be considered. In addition, scenarios where multiple warnings prior to an event occur make calculating traditional performance indices such as false positive rate and sensitivity not straight forward and intuitive. In presence of such scenarios, researchers may only count the warning closest to an event disregarding the remainder of warnings on the record [7] or sometimes look at the performance metric as a function of time [8].

We propose an approach which estimates the above three characteristics of a warning system considering multiple warnings per record, warning timeliness and warning burden (occurrence of multiple warning per event/record) [8]. The method involves identifying the time before an event of interest when warnings are meaningful and evaluating how frequently warnings occur before, within, and after those times. We previously demonstrated the concept using an enriched data set where the event of interest was known to occur for every experiment (i.e., the dataset used for our example analysis lacked non-event records and thus non-events falsely detected as events). While we discussed the need and mechanism to incorporate non-events falsely detected as events in the framework, it was not demonstrated. Here, we provide information on how the proposed framework can be implemented with datasets including both events and non-events (control cases). The event records use the same data presented in our previous study using data from a hemorrhage-induced hypotension animal investigation [9]. We have supplemented this dataset with non-event records from a study with similar experimental protocol as our previously reported hemorrhage study but from baseline periods without interventions inducing hypotension [10].

### Framework to characterize performance

**Fig. 1:**
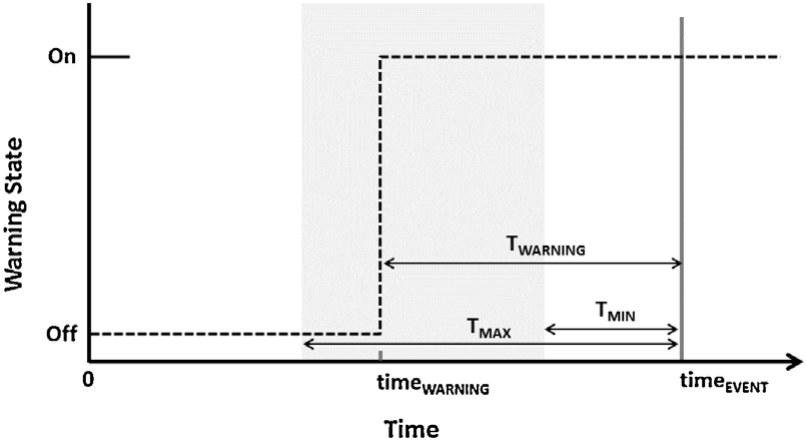
Fiducial points used to characterize a warning. Dashed lines indicate the warning state when time of critical event is time_EVENT_ and warning onset is time_WARNING_. T_WARNING_ is defined as the time from the warning onset (time_WARNING_) to time of critical event (time_EVENT_). The gray box bounded by T_MAX_ and T_MIN_, indicates the maximum and minimum amount of time prior to the event when a warning is considered meaningful. T_MIN_ and T_MAX_ should be selected based on the event of interest that the warning system will be used for. For the non-event (control) data, time_EVENT_ is undefined, while for events where a warning was absent (missed) time_WARNING_ is undefined.

The framework is calculated using following fiducial time points which characterize a warning (identified on Fig. 1):

• time_EVENT_ – time of the critical health event
• time_WARNING_ – time of warning onset
• T_WARNING_ (=time_EVENT_ − time_WARNING_) – time from the warning onset (time_WARNING_) to time_EVENT_
• T_MAX_ – maximum amount of time prior to the event when a warning is expected to be meaningful
• T_MIN_ – minimum amount of time prior to the event that would allow for meaningful action (depending on the application T_MIN_ may be 0, the time of the event, or negative – i.e. after the event)

T_MAX_ and T_MIN_ are pre-specified limits which are maintained in the code as constants. These should be defined by the user based on subject matter expertise for what is meaningful for a given application. Fiducial points, time_EVENT_ and time_WARNING_ are calculated for each detected event on each event/non-event record which is included in the input dataset to the code as a csv file. The code to create the proposed framework and datasets for the case study (see Section Method demonstration) are provided as Supplementary materials. To implement the framework, time-series data with an annotation of critical event time, or lack of event time, for each subject should be first converted to a binary array where 1 indicates a warning is active and 0 that a warning is not active. Datasets containing fiducial points for each warning are then extracted from this binary array. All time series records should be of similar length in order to avoid misleading results.

Two charts are calculated to estimate three performance metrics. The first chart (time profile of warning proportions) estimates 1) probability a critical health event will occur when warning is given (analogous to positive predictive value). The second chart (time profile of warnings per records) estimates, 2) proportion of warned events to all events (analogous to sensitivity) and 3) proportion of warned non-events to all non-events (analogous to false positive rate). These charts also provide information about the timeliness of the warnings with respect to event occurrence and the warning burden of the system (which represents multiple warning occurrence for a single record) that are not captured directly by traditional performance metrics calculated considering each record as a single true/false positive/negative.

Based on T_WARNING_, T_MIN_ and T_MAX_, each warning is categorized into one of five bins:

• False: Warnings from non-event records (time_EVENT_ undefined)
• Early: Warnings from event records where T_WARNING_ ≥ T_MAX_
• On time: Warnings from event records where T_MAX_ > T_WARNING_ ≥ T_MIN_
• Late: Warnings from event records where T_WARNING_ < T_MIN_
• Missed: Event records where a warning was not generated (time_WARNING_ undefined)

Given a dataset, the number of warnings in each of the above five bins are normalized two ways to look at the data with respect to two unit of analyses: “per warning” and “per record”.

1 Normalize with total number of warnings to generate time profile of warning proportions

The number of warnings in each bin are divided by the sum of all warnings. This chart estimates probability a critical health event occurs in time ε [T_MIN,_ T_MAX_] when a warning is given (i.e. unit of analysis is “per warning”). On this bar chart with 5 bins (in the order of False, Early, On Time, Late, Missed), an ideal warning system will have bin On Time = 1 with the other four bins = 0. Frequent early or non-event warnings will result in the distribution having a thicker right tail (bins False and/or Early > 0). Frequent late or missed warnings creates a thicker left tail (bins Late and/or Missed > 0). When there are multiple warnings per record, the sum across all five bins will be greater than 1.

2 Normalize with respect to total number of records in the corresponding record group to generate time profile of warnings per records

To accurately obtain a unit of analysis of “per record”, the number of False warnings is divided by the total number of non-event records, and the number of Early, On Time, Late and Missed warnings are divided by the total number of event records. This chart estimates the proportion of warned events occurring in time ε [T_MIN,_ T_MAX_], the proportion of warned non-events, and the warning burden. An ideal warning system will have a distribution with bin On Time = 1 while other four bins = 0. When a system warns events frequently on time, bin On Time will have a taller bar compared to other four bins. When a system warns on non-events, bin False > 0. The warning burden is captured multiple ways in this chart. The sum across the last four bins (Early, On Time, Late and Missed) > 1 indicates multiple warnings per single event. If there are multiple events with timely warnings while some events have missed, early or late warnings, bin On Time = 1 while bins Missed, Early or Late ≠ 0.

### Method demonstration

#### Experimental approach

We previously presented an example of using the proposed framework for event records in [8] using an experimental animal study of hemorrhage. Here, we extend that presentation by also including non-event records.

##### Event records:

Experiments were performed at the University of Texas Medical Branch under a protocol reviewed and approved by the Institutional Animal Care and Use Committee (IACUC) and conducted in compliance with the guidelines of the National Institutes of Health and the American Physiological Society for the care and use of laboratory animals. For the first experiment, as described in detail in [9] and summarized in [8], 8 female sheep underwent two hemorrhages on different days (one day at 1.25 mL/kg_BW_/min and the other at 0.25 mL/kg_BW_/min). The hemorrhage was performed until a hypotensive event occurred that was defined as a drop in mean arterial pressure (MAP) of 30 mmHg. Continuous physiological monitoring including arterial blood pressure was performed and recorded through the baseline (at least 30 min), hemorrhage, and post-hemorrhage periods. The arterial pressure recordings were analyzed for this study as described in [8].

##### Non-event records:

The second experiment was performed with IACUC approval from the same facility as the first one [10]. Twelve adult female sheep were used to study progression of physiologic instabilities during pneumonia-induced sepsis. Physiological variables including arterial blood pressure were continuously recorded during sepsis as well as prolonged baseline periods (24 h) from each sheep using the same instruments as hemorrhage study. Arterial blood pressure signals from randomly selected 2 h segments during the baseline period (after excluding first 12 h to avoid periods of acclimatization) were analyzed similar to event records to create non-event records. These were used to represent hemodynamically stable periods without hypotensive events. The data length of 2 h was selected to approximate the length of the longest event records.

##### Normalized shock index as a warning indicator:

Data analysis for both event and non-event records was performed as described in [8]. Shock Index [heart rate (HR)/systolic blood pressure (SBP)] was computed by extracting HR and SBP from the arterial pressure signal. This was converted to an index on a 0–1 scale termed the normalized Shock Index (SI_NORM_) for this work. To be consistent with our previous study, population baseline mean and standard deviation of the event records (hemorrhage cases) was computed and SI_NORM_ was estimated from the Cumulative Distribution Function (CDF) for a normal distribution with the event records baseline mean and standard deviation. A warning was defined when SI_NORM_ ≥ 0.9. Two configurations of the warning system were considered as previously described. RESPONSIVE treated the warnings as a straight binary interpretation of SI_NORM_ with a warning on when SI_NORM_ was greater than 0.9 and off whenever it was below 0.9. STAY-ON remained on once SI_NORM_ went above 0.9 until SI_NORM_ was lower than 0.9 for 10 min. The two warning strategies, RESPONSIVE and STAY-ON, were designed to have different numbers of warnings due to the latching configuration, STAY-On strategy groups transient responsive alerts, hence reducing the total number of warnings (see Fig. 3 on [8] for a graphical illustration).

As previously described, time_EVENT_ was defined as the time when MAP decreased below 30 mmHg MAP from baseline (i.e., stopping criteria for the hemorrhage study). T_MIN_ was set to 1 min, and T_MAX_ was set to 14 min [8] which are maintained as constants in the Supplementary material R code. In the Supplementary material datasets, for non-event records, time_EVENT_ remained undefined (coded as missing i.e. NA in the provided csv dataset for R code) while for event records where a warning was not generated time_WARNING_ remained undefined (coded as missing i.e. NA in the provided csv dataset for R code).

#### Results and description of characterization

Positive predictive value, sensitivity, and false positive rate (false positive rate = 1 - specificity) are shown in Table 1 for the two warning system designs. We calculated these traditional performance metrics considering a warning state on at any point within [T_MAX_, T_MIN_] as a true positive on event records and at least one warning at any point of the non-event record as a (false) positive (i.e., only a single false positive was counted for each animal).

**Table 1:** Traditional performance metrics for RESPONSIVE and STAY-ON warning system designs.

		RESPONSIVE	STAY-ON
No. of records (Median, minimum – maximum record length in minutes)	Event (Positive)	16 (24, 7–114)	
	Non-event (Negative)	12 (120, 120–120)	
No. event records with warning state on at any point within [T_MAX_, T_MIN_]		14	14
No. of non-event records with at least one warning		5	5
Positive Predictive Value		73.7 (14/19)	73.7 (14/19)
Sensitivity		87.5 (14/16)	87.5 (14/16)
False Positive Rate (False Positive Rate = 1 – Specificity)		42.7 (5/12)	42.7 (5/12)

**Fig. 2:**
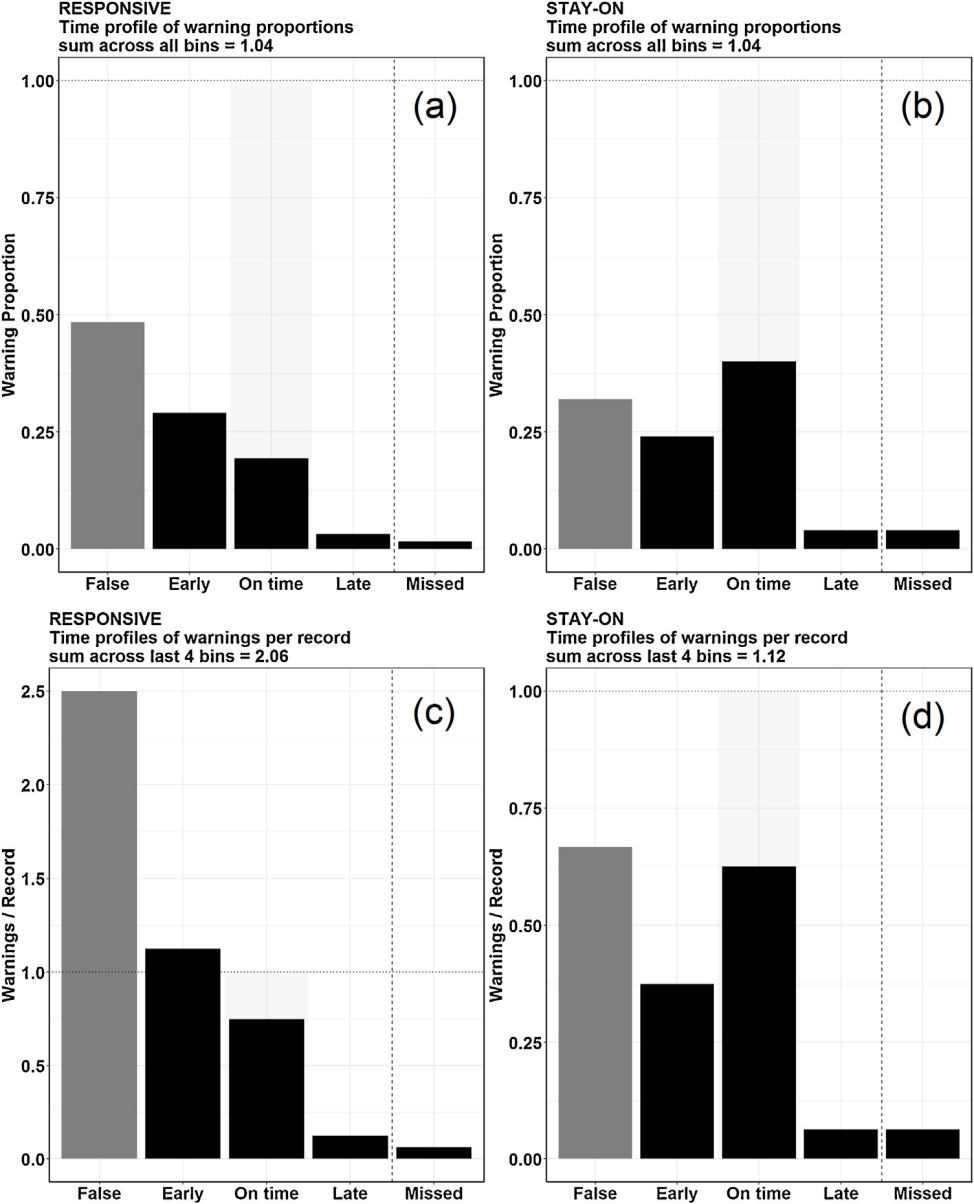
(a) and (b) Time proﬁle of warning proportions, for RESPONSIVE and STAY-ON designs respectively. (c) and (d) Time proﬁle of warnings per record for RESPONSIVE and STAY-ON designs respectively.

The results generated by applying the proposed framework to the RESPONSIVE and STAY-ON warning system designs are shown in Table 2 and Fig. 2. The proposed framework takes into account every warning on both event records and non-event records.

**Table 2:** The proposed framework applied for RESPONSIVE and STAY-ON warning system designs.

	Warning Category	No. of Warnings	Total No. of Warnings	No. of Records	Warning Proportion (No. of Warnings/Total No. of Warnings)	Warnings per record (No. of Warnings/No. of Records)
RESPONSIVE	*False*	30	62	12	0.48	2.5
	*Early*	18		16	0.29	1.12
	*On Time*	12			0.19	0.75
	*Late*	2			0.03	0.12
	*Missed*	1			0.02	0.06
STAY-ON	*False*	8	25	12	0.32	0.67
	*Early*	6		16	0.24	0.38
	*On Time*	10			0.40	0.62
	*Late*	1			0.04	0.06
	*Missed*	1			0.04	0.06

As noted in our previous work [8], the **time profile of warning proportions** (Fig. 2a and b) shows that a given warning generated by STAY-ON is more likely to be On Time (within [T_MAX_, T_MIN_]) than one generated by RESPONSIVE. Fig. 2b shows that ˜75% of all RESPONSIVE warnings were either false or occurred before T_MAX_ (sum across bins False and Early = 0.77), and this is ˜50% (sum across bins False and Early = 0.56) for the STAY-ON design (Fig. 2b).

The **time profile of warnings per record** estimator (Fig. 2c and d) shows that the STAY-ON design is more likely to correctly warn of true events occurring in time ε [T_MIN,_ T_MAX_] (warning frequently within the predefined window and less likely to warn non-events as events, demonstrated as shorter bar for bin False) compared to the RESPONSIVE design. The larger warning burden of RESPONSIVE design is evident on time profile of warnings per record with a large >1 sum across the last four bins compared to STAY-ON design (ratio of all warnings to all records of 2.06 for RESPONSIVE compared to 1.12 for STAY-ON).

Due to the natural difference of format of the two types of metrics (i.e., traditional metrics are point estimates while the proposed metrics are distributions), it is not possible to do a direct comparison using a statistical test but a qualitative comparison. The proposed framework considers the timing of warnings based on what is meaningful for a given application. It also provides a simple graphical display of the results to aid interpretation. While the two strategies, RESPONSIVE and STAY-ON, were designed to have different numbers of warnings due to the latching configuration, the calculated traditional performance metrics showed similar performance between two alarm strategies (Table 1). The proposed framework contrasts the two strategies on multiple aspects: 1) time profile of warning proportions shows that warnings generated by STAY-ON are more likely to be on time when compared to RESPONSIVE which is more likely to have warnings be false or early and 2) RESPONSIVE was more likely to warn for non-events, warn early for true events and overall generate a high warning burden compared to STAY-ON design (as shown by time profile of warnings per record).

One limitation of the framework is when estimating the proportion of warned non-events to non-event records, **time profile of warnings per records** fails to differentiate whether multiple false alarms came from the same non-event record or different non-event records. This, however, is not the case when estimating the proportion of warned events to event records. That is because **time profile of warnings per records** shows similar heights on the bar On Time, while the heights of the other three bars (Early, Late and Missed) can be used to estimate whether all On Time warnings came from different event records or same event record multiple times. If coming from the same event record multiple times, bins Missed, Early or Late will have bars greater than 0 and there will be a larger than 1 sum across the last four bins (Early, On Time, Late, Missed) compared to the former.

The study design and dataset construction are critical factors that will affect the reported performance using this, or any, performance assessment approach for an early warning index. To make the charts presented in this framework most meaningful, the prevalence of the event of interest should reflect the prevalence in clinical scenarios and the length of time series should be equal for all records. In the current study, we present results with a mix of positive and negative cases to demonstrate the approach but did not attempt to match the prevalence with a clinical scenario.

### Conclusion

In this article, we present how our previously reported framework could be extended with non-event records to provide a more complete characterization of warning index performance, by estimating 1) probability a critical health event will occur when warning is given (analogous to positive predictive value), 2) proportion of warned events to all events (analogous to sensitivity) and 3) proportion of warned non-events to all non-events (analogous to false positive rate). This framework also provides information about the timeliness of the warnings with respect to event occurrence and the warning burden of the system. Using a case study of two warning strategies on a dataset curated to incorporate both event and non-event records, we demonstrate how our previously proposed framework for evaluating early warning indices can be used to characterize performance of early warning indices.

## Disclosures

This article reflects the views of the authors and should not be construed to represent the FDA’s views or policies. The mention of commercial products, their sources, or their use in connection with material reported herein is not to be construed as either an actual or implied endorsement of such products by the Department of Health and Human Services.
