# Supplementary material for: A framework to characterize the performance of early warning index alarm systems for patient monitoring
Source: MethodsX. 2019 Jul 8;6:1660–7. doi: 10.1016/j.mex.2019.07.003 (PMC6660561; doi:10.1016/j.mex.2019.07.003)
Supplement: Supplementary file 4 [file mmc4.pdf]

Calculate Early Warning Index Performance

Chathuri Daluwatte, PhD Chathuri.Daluwatte@fda.hhs.gov, cldaluwatte@mail.missouri.edu

July 2018

Table of Contents

Abstract 2
References 2
Load libraries and declare utility functions 2
Load and prepare the data for analysis 2
  Load dataset and set $Tmin$ and $Tmax$. 3
  Calculate $Twarning$ and warning category. 3
Proposed Early Warning Index Performance Metrics 4
  Time profile of warning proportions 5
  Time profiles of warnings per record 6

LICENSE

Author - Chathuri Daluwatte, PhD

This code is free software: you can redistribute it and/or modify it under the terms of the GNU General Public License as published by the Free Software Foundation, either version 3 of the License (GNU GPL3), or (at your option) any later version.

This code is distributed in the hope that it will be useful, but WITHOUT ANY WARRANTY; without even the implied warranty of MERCHANTABILITY or FITNESS FOR A PARTICULAR PURPOSE. See the GNU General Public License for more details.

You should have received a copy of the GNU General Public License along with this program. If not, see here.

DISCLAIMER

This code does not necessarily reflect any position of the Government or the Food and Drug Administration.

This code and documentation were developed by the author in her capacities as Oak Ridge Institute for Science and Education (ORISE) research fellow at the U.S. Food and Drug Administration (FDA).

FDA assumes no responsibility whatsoever for use by other parties of the Software, its source code, documentation or compiled executables, and makes no guarantees, expressed or implied, about its quality, reliability, or any other characteristic. Further, FDA makes no representations that the use of the Software will not infringe any patent or proprietary rights of third parties. The use of this code in no way implies endorsement by the FDA or confers any advantage in regulatory decisions.

Abstract

This script generates time profile of warning proportions and time profiles of warnings per record based on analysis published in Daluwatte et al. (2019). Data used for this study are available as supplementary material in Daluwatte et al. (2019).

References

1. Daluwatte C, Yaghouby F, Scully CG. A framework to characterize the performance of early warning index alarm systems for patient monitoring. MethodsX. 2019.
2. Scully CG, Daluwatte C. Evaluating performance of early warning indices to predict physiological instabilities. Journal of Biomedical Informatics. 2017.

Load libraries and declare utility functions

```
############################################################################
#Load required libraries
############################################################################

## Graphics and data wrangling
library(tidyverse);
library(pander);
library(ggplot2);
library(gridExtra);
library(flextable);
library(officer);
textsize = 20
```

Load and prepare the data for analysis

Input to this program consists of a csv file with following columns. Each line corresponds to one warning.

- id: record indicator
- time_warning: time of the warning onset from time zero
- time_event: time of the event from time zero
- iscontrol: indicate if the record is with or without event. 1 if recocrd is without event 0 otherwise

Warnings from records with an event should be added with:

- isconrol set to 0
- time_warning set to the warning onset from time zero
- time_event set to time of the event from time zero

Warnings from records without an event should be added with:

- iscontrol set to 1
- time_warning set to the warning onset from time zero
- time_event set to missing

Records with an event which didn't trigger a warning should be added with:

- iscontrol set to 0
- time_warning set to missing
- time_event set to time of the event

Records without an event which didnt trigger a warning should be added with:

- iscontrol set to 1
- time_warning set and time_event set to missing

Load dataset and set $Tmin$ and $Tmax$.

As defined in Daluwatte et al. (2019) and Scully and Daluwatte (2017), $Tmin$ and $Tmax$ is the interval prior to event where a warning should be considered meaningful. $Tmax$ is the longest duration of notice prior to an event that a warning index was designed to provide warning. $Tmin$ is the shortest duration of notice prior to an event that a warning index was designed to provide warning .

```
## Load dataset.
data <- read.csv("data_stayon.csv",as.is = T, stringsAsFactors = F)
tmax = 14
tmin = 1
```

Calculate $Twarning$ and warning category.

- True negatives: iscontrol = 1 & time_warning == NA & time_event == NA
- False positives (alarms in control data): iscontrol = 1 & time_warning != NA & time_event == NA
- False negatives (missed alarms): iscontrol =0 & time_warning = NA & time_event != NA
- True Positives (early): iscontrol = 0 & twarning >= tmax
- True Positives (on time): iscontrol = 0 & twarning < tmax & twarning >= tmin
- True Positives (late): iscontrol = 0 & twarning < tmin & twarning >= 0

```
## Calculate no. of control events
ncontrol <- nrow(data %>% filter(iscontrol == 1) %>% select(c(id)) %>%
distinct())
```

```
## Calculate no. of positive events
nevent <- nrow(data %>% filter(iscontrol == 0) %>% select(c(id)) %>%
distinct())
## Calculate no. of alarms
nalarm <- nrow(data %>% filter(!is.na(time_warning)) %>%
select(c(id,time_warning)) %>% distinct())

## Calculate Twarning and alarm category
data <- data %>% mutate(twarning = time_event - time_warning,
                        alarmcat = ifelse(iscontrol == 1 &
is.na(time_warning),"true negative",{
                         ifelse((iscontrol == 1 &
!is.na(time_warning)),"false",{
                          ifelse((iscontrol == 0 & is.na(time_warning) &
!is.na(time_event)),"missed",{
                           ifelse((iscontrol == 0 & twarning >=
tmax),"early",{
                            ifelse((iscontrol == 0 & twarning < tmax &
twarning >= tmin),"on time",{
                             ifelse((iscontrol == 0 & twarning <
tmin),"late","")
                            })
                           })
                          })
                         })
                        }))
```

Proposed Early Warning Index Performance Metrics

```
## Calculate time profile of warning proportions and time profiles of
warnings per event
alarmcount <- data %>% group_by(alarmcat) %>%
  summarise(count = n()) %>% ungroup() %>%
  filter(alarmcat != "true negative") %>%
  mutate(nevent = ifelse(alarmcat == "false", ncontrol, nevent),
         nalarm = nalarm,
         alarmcat = factor(alarmcat,levels = c("false","early","on
time","late","missed"),
                            labels = c("False","Early","On
time","Late","Missed")),
         propofwarn = count/nalarm,
         warnperevent = count/nevent) %>% arrange(alarmcat)

alarmcount.formatted <- flextable(alarmcount %>% mutate(propofwarn =
round(propofwarn,2),
                                                          warnperevent =
round(warnperevent,2)))
alarmcount.formatted <- width(alarmcount.formatted,width=0.75)
alarmcount.formatted <- set_header_labels(alarmcount.formatted,
```

```
                                      alarmcat = "Warning Category",
                                      count = "No. of Warnings",
                                      nevent = "Total No. of Records",
                                      nalarm = "Total No. of Warnings",
                                      propofwarn = "Warning proportion \n(No.
of Warnings/Total No. of Warnings)",
                                      warnperevent = "Warnings per record
\n(No. of Warnings/Total No. of Records)")
alarmcount.formatted <- merge_v(alarmcount.formatted, j =
c("nevent","nalarm"))
alarmcount.formatted
```

Warning Category	No. of Warnings	Total No. of Records	Total No. of Warnings	Warning proportion (No. of Warnings/Total No. of Warnings)	Warnings per record (No. of Warnings/Total No. of Records)
False	8	12		0.320	0.670
Early	6			0.240	0.380
On time	10	16	25	0.400	0.620
Late	1			0.040	0.060
Missed	1			0.040	0.060

```
# write to word
alarmcount.formatted.doc <- read_docx()
alarmcount.formatted.doc <- body_bookmark(alarmcount.formatted.doc,'Early
Warning Performance Metrics')
alarmcount.formatted.doc <-
body_add_flextable(alarmcount.formatted.doc,alarmcount.formatted)
tmp <- print(alarmcount.formatted.doc,'./Early Warning Performance
Metrics.docx')
```

Time profile of warning proportions

```
## Plot time profile of warning proportions
p1<- ggplot(alarmcount,aes(x = alarmcat, y = propofwarn, fill = alarmcat)) +
  geom_bar(stat="identity") +
  annotate("rect", xmin = 2.5, xmax = 3.5, ymin = 0, ymax = 1,
           fill = "grey70",  alpha = .1) +
  geom_bar(stat="identity") +
  scale_fill_manual(values =
c("grey50","grey0","grey0","grey0","grey0"),guide = F) +
  geom_vline(xintercept = 4.5, linetype = "dashed") +
  geom_hline(yintercept = 1, linetype = "dotted") +
  theme_bw() +
```

```
  ggtitle('Time profile of warning proportions') +
  ggtitle(paste("Time profile of warning proportions \n","sum across all bins
= ",round((alarmcount %>%

summarize(sum = sum(propofwarn)))$sum,2), sep = "" )) +
  ylab('Warning Proportion') + xlab('')
print(p1)
```

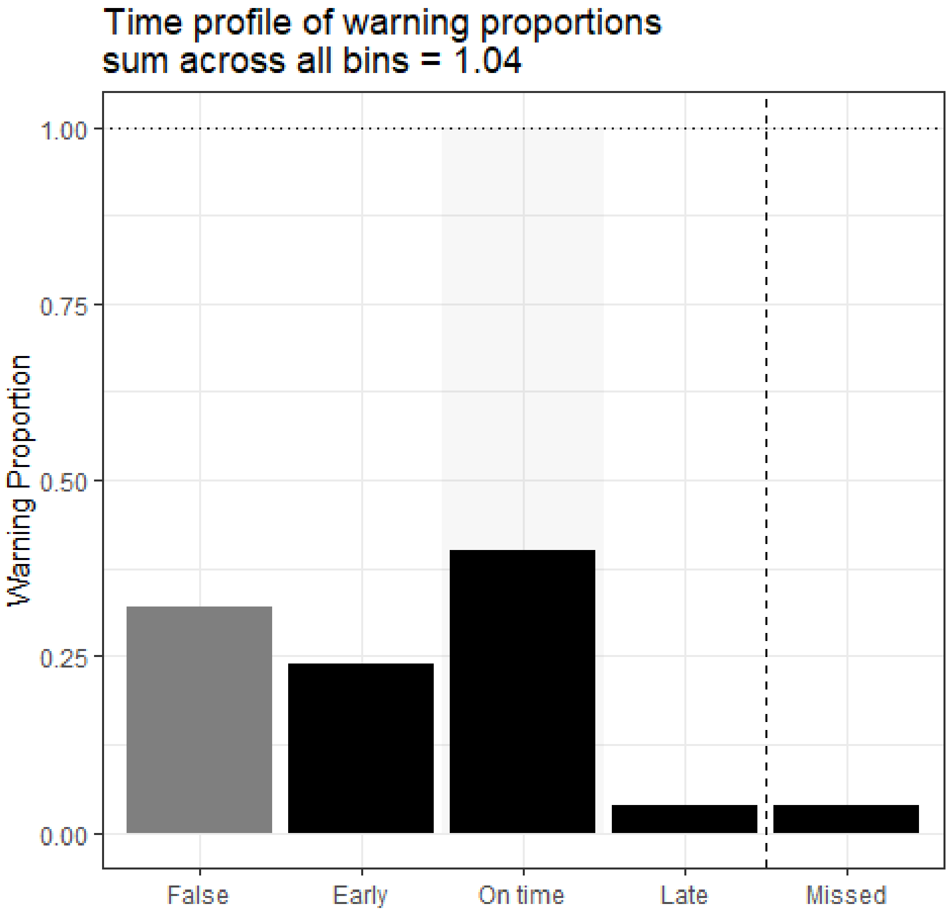

Time profiles of warnings per record

```
## Plot time profiles of warnings per record
p2 <- ggplot(alarmcount,aes(x = alarmcat, y = warnperevent, fill = alarmcat))
+
  geom_bar(stat="identity") +
  annotate("rect", xmin = 2.5, xmax = 3.5, ymin = 0, ymax = 1,
           fill = "grey70",  alpha = .1) +
  geom_bar(stat="identity") +
  scale_fill_manual(values =
c("grey50","grey0","grey0","grey0","grey0"),guide = F) +
  geom_vline(xintercept = 4.5, linetype = "dashed") +
  geom_hline(yintercept = 1, linetype = "dotted") +
  theme_bw() +
```

```
  ggtitle(paste("Time profiles of warnings per record \n","sum across last 4 
bins = ",round((alarmcount %>%
                                                  filter(alarmcat != "False")
%>%
                                                  summarize(sum = 
sum(warnperevent)))$sum,2), sep = "" )) +
  ylab('Warnings / Record') + xlab('')
print(p2)
```

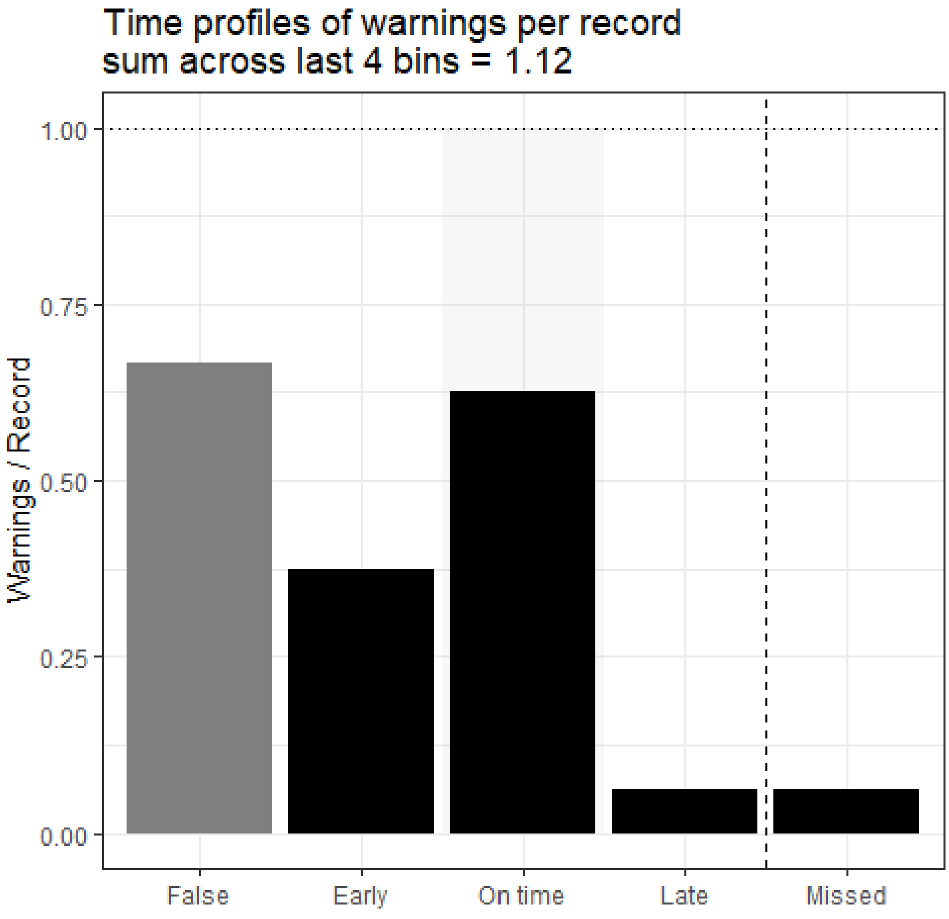


```
figurename <- paste0('EarlyWarningIndexPerformance','.png')
p <- png(figurename,width = 400, height = 500, units = 'mm', res = 150)
p1 <- p1 +
  theme(axis.text =element_text(size=textsize,face="bold",angle = 0,hjust = 
1),
        axis.title=element_text(size=textsize,face="bold"),
        axis.line = element_line(size = 0.5),
        plot.title = element_text(size=textsize,face="bold"),
        plot.margin= unit(c(0.1,0.1,0.1,0.1),'cm'),
        panel.border = element_rect(fill = NA,colour = "black", size = 1),
        panel.spacing = unit(0.1, "cm"),
        strip.text = element_text(size = 30,face="bold"))
```

```
p2 <- p2 +
  theme(axis.text =element_text(size=textsize,face="bold",angle = 0,hjust =
1),
        axis.title=element_text(size=textsize,face="bold"),
        axis.line = element_line(size = 0.5),
        plot.title = element_text(size=textsize,face="bold"),
        plot.margin= unit(c(0.1,0.1,0.1,0.1),'cm'),
        panel.border = element_rect(fill = NA,colour = "black", size = 1),
        panel.spacing = unit(0.1, "cm"),
        strip.text = element_text(size = 30,face="bold"))
grid.arrange(p1,p2,nrow = 2,ncol = 1)
p<-dev.off()
```
